# Multiple-Exposure Image Fusion for HDR Image Synthesis Using Learned Analysis Transformations †

**DOI:** 10.3390/jimaging5030032

**Published:** 2019-02-26

**Authors:** Ioannis Merianos, Nikolaos Mitianoudis

**Affiliations:** Electrical and Computer Engineering Department, Democritus University of Thrace, 67100 Xanthi, Greece; nmitiano@ee.duth.gr

**Keywords:** image fusion, exposure fusion, independent component analysis (ICA)

## Abstract

Modern imaging applications have increased the demand for High-Definition Range (HDR) imaging. Nonetheless, HDR imaging is not easily available with low-cost imaging sensors, since their dynamic range is rather limited. A viable solution to HDR imaging via low-cost imaging sensors is the synthesis of multiple-exposure images. A low-cost sensor can capture the observed scene at multiple-exposure settings and an image-fusion algorithm can combine all these images to form an increased dynamic range image. In this work, two image-fusion methods are combined to tackle multiple-exposure fusion. The luminance channel is fused using the Mitianoudis and Stathaki (2008) method, while the color channels are combined using the method proposed by Mertens et al. (2007). The proposed fusion algorithm performs well without halo artifacts that exist in other state-of-the-art methods. This paper is an extension version of a conference, with more analysis on the derived method and more experimental results that confirm the validity of the method.

## 1. Introduction

The recent technological progress in imaging sensors development has triggered the introduction of image cameras with very high specifications. Nonetheless, the problem of capturing natural scenes, in the same quality as captured by the human visual system, is far from being solved with today’s technology. Imaging sensors in modern cameras feature maximum-to-minimum light intensity of approximately $2^{8}$–$2^{17}$. The maximum-to-minimum light intensity ratio is termed *dynamic range*. The logarithm of the dynamic range with base 2 is measured in *stops*. A commercial DSLR camera can capture scenes with 8–12 stops dynamic range. On the other hand, professional movie cameras, such as the Red Monstro (https://www.red.com/dsmc2), can capture scenes with approximately 17 stops. The dynamic range of natural-world scenes exceeds 24 stops. The human visual system can very efficiently capture and comprehend such content. If we use a modern imaging sensor to capture such a high-dynamic-range scene, the result will often be that several areas of this image will be either under-exposed or over-exposed. This implies that the captured image will feature darker or whiter colors than in real life or even some parts may be completely non-visible. Thus, information from the natural scene cannot be captured in full. Typical scenes that feature HDR are sunset or sunrise scenes or plain daylight scenes with objects under shadows. Another field, where modern imaging can improve to increase the dynamic range of image acquisition and storage, is the color bit depth, i.e., the number of bits attributed to each color. The common format is 8 bits/color, i.e., 24 bits/pixel for RGB representation, which is typical for a Low-Dynamic Range (LDR) photo that can be viewed on normal displays. To increase the dynamic range that can be captured by modern devices, the color bit length needs also to be increased to create High-Dynamic Range (HDR) imaging.

A possible solution to this shortcoming is to use *multi-bracketing photography*. In other words, we capture the scene using a common low-dynamic-range imaging sensor, nonetheless, multiple photos are taken at different exposures. Thus, areas of different dynamic range can then be captivated in one (or maybe more) of the different exposures. Thus, it is important to choose the different exposures at which the camera should operate, to capture the dynamic range of a natural scene. In most proposed setups, three exposures (one normal exposure, one below-normal and one above-normal exposure) are commonly adequate to capture the most demanding high-dynamic-range images. Of course, one can use more exposures, but this is increasing the computational complexity of the framework. Once this is done, the obvious problem is to create a fused image that combines the useful visual information from all input images. The fused image should resemble the image attained by the human visual system as accurately as possible. In the seminal work of Devebec and Malik [1], a method is introduced which can fuse the multiple exposures into a single, high-dynamic-range radiance map whose pixel values are proportional to the true radiance values in the scene. The problem is that these high-dynamic-range maps cannot be viewed on normal LDR displays or printing devices. Therefore, a tone-mapping approach is usually applied to compress the HDR content back to LDR content to be visible to normal displays [2]. It should be stressed that the final LDR content is superior to any of the original LDR input exposures. Instead of creating an HDR representation and then returning to LDR with tone mapping, an alternative approach was proposed, where the three exposures are combined to form an LDR representation with improved dynamic range [3]. This problem is often addressed by *image-fusion* algorithms [4]. In the case of multiple-exposure inputs, the term *exposure fusion* is often encountered in the literature [3], to describe this problem.

*Multiple-Exposure Fusion* (MEF) has recently attracted the attention of many researchers. Bogoni and Hansen [5] proposed a pyramid-based pattern-selective fusion scheme. Luminance and chrominance components were decomposed into Laplacian and Gaussian pyramids, respectively. However, the color scheme in the fused image tended to be very close to the average color image. This was because colors with saturation closest to the average saturation were selected for the fused image. In [3], Mertens et al. introduced a MEF scheme, where the input images are initially decomposed into the Laplacian pyramid. The decomposed images are then combined using fusion weights derived from three different factors: contrast, saturation, and well-exposedness. In [6], Vonikakis et al. proposed a MEF scheme, where an Illumination Estimation filtering method is employed to estimate well-exposedness of the scene. The method employs these first estimates to derive fusion weights via fuzzy membership functions that will transfer pixels of medium exposure to the produced fused image. In [7], Tico et al. used photometric calibration to address the motion-blur problem that may be encountered in longer exposures. In [8], Jinho and Okuda introduced novel weighting fusion functions, which retain their independence on different exposure areas with little overlap. Maximum a posteriori (MAP) estimation is also employed to address motion-blur and occlusions problems. In [9], Shen et al. introduced a framework that used generalized random walks to estimate a globally optimal solution that is constrained by two quality measures, i.e., local contrast and color consistency. Their approach formulates the fusion task as a probability estimation problem. In [10], Li and Kang proposed a weighted sum-based multi-exposure image-fusion method consisting of two stages: (a) three image features, i.e., local contrast, brightness, and color dissimilarity are first estimated to construct weight maps, which are then refined by recursive filtering, (b) the fused image is constructed by the weighted sum of source images.

In the literature, we can also encounter more general image-fusion methods. In [11], Mitianoudis and Stathaki introduced a transform-domain image-fusion framework based on Independent Component Analysis (ICA). Instead of using common transforms, the novelty was they used a transformation that was estimated from a group of similar-content images [11]. In addition, several novel fusion rules were introduced in [11,12]. The proposed method operates, as follows. First, the input images are segmented into small local patches. The patches are then moved to the ICA-transform domain using the estimated ICA bases, where the patches are fused using a fusion rule and then are moved to the spatial domain again. The local patch means are subtracted before moving to the ICA domain and are stored to be used for the reconstruction of the fused image. An average of the stored means for the patch along all input images usually works well for “out-of-focus” fusion and is used to reconstruct the fused image [11]. However, in the case of multimodal inputs, Mitianoudis and Stathaki [4] proposed an update rule that maximizes the Piella and Heijmans Fusion Quality index [13] to estimate an optimal mean value for each patch of the fused image. In [14], Farid et al. presented a novel method to perform out-of-focus image fusion by Content Adaptive Blurring. More specifically, they estimate the local image quality in neighborhoods and pixels with focused content are filtered with non-uniform blur. The segmentation map that is produced is further refined with morphological operators and graph-cut techniques to produce the final segmentation/fusion map. Finally, in transform-domain fusion, we had the emergence of sparse representation techniques over the last years. This is a step further from the ICA bases, used by Mitianoudis and Stathaki [4], since the objective is to estimate overcomplete/redundant bases that will lead to sparser representations, i.e., better signal energy compaction. These were initially used for image denoising, with the example of K-SVD [15]. Sparse representations have also been used in out-of-focus and multimodal image fusion. A complete review on this topic can be found in [16].

In this work, we propose a multiple-exposure fusion algorithm, where the luminance (intensity) channels are fused via the ICA-based image-fusion approach of [4], while the chrominance channels are fused using the Mertens et al. method [3]. The output will be a normal TrueColor (24 bit-RGB) image with increased dynamic range from multiple exposures. A conference version of this work was presented here [17]. This paper is an extended version with all contents revisited and extended. More specifically, all the theoretical analysis and description of the proposed system has been extended with more details and analysis. The description of the ICA fusion part along with the corresponding fusion rules has been extended on the training and the actual procedure of performing ICA fusion. The description of each of the post-processing modules has been extended in more detail. The experimental section now includes more examples. We added the method of Li and Kang [10] for comparison. We highlighted the existence of halo artifacts in some of the methods with zoomed figures, to visualize the original claims. We added another important experiment to highlight the improved structural integrity of the proposed method. Another image set was also added for visual comparison.

## 2. The Proposed Multiple-Exposure Image-Fusion System

**Figure 1:**
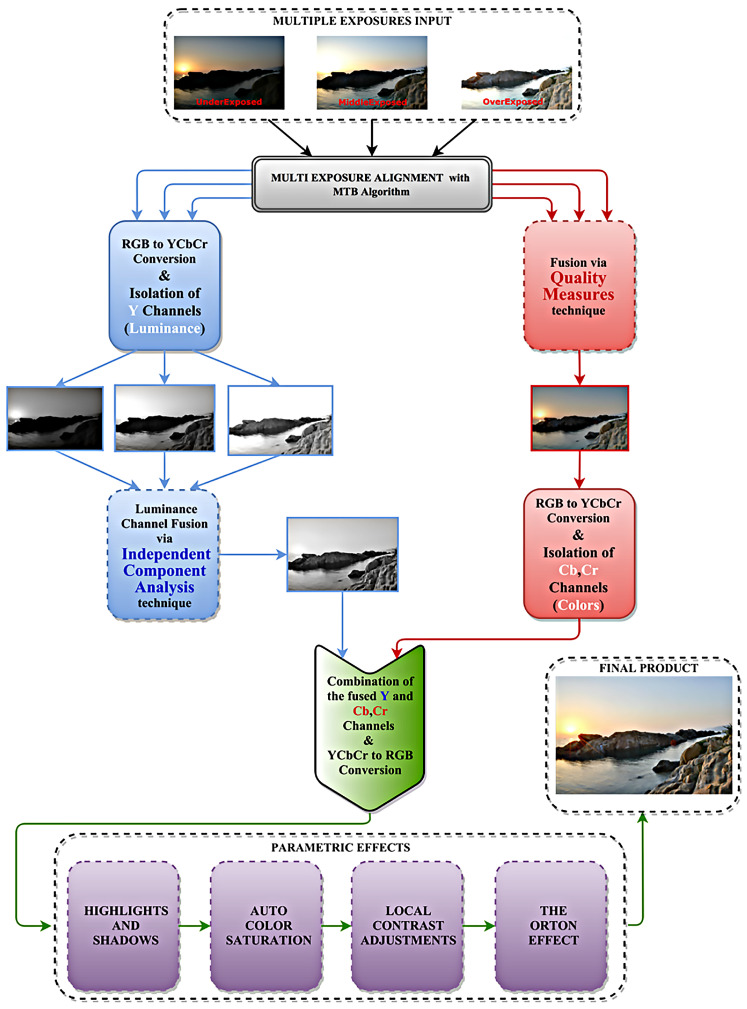
The proposed Exposure Fusion system.

The proposed multiple-exposure image-fusion approach is segmented into several operational block. The whole system is depicted in Figure 1. In the following paragraphs, we discuss its individual system blocks.

### 2.1. Image Alignment—Color System Conversion

In the proposed system, the three-exposure-image setup (under-exposed, normally exposed, over-exposed) was employed as in the majority of MEF systems. Nonetheless, the proposed system can function with any number of exposure inputs without any further modification. The first stage in the system was to examine the three input images for possible mis-registrations. The *Median Threshold Bitmap* (MTB) method of Ward [18] was used in our system to tackle the problem. The MTB method addresses translational registration errors only and is not computationally prohibitive. The assumption of translational registration errors is important; however, this will be most probably the case in real-life situations. Since the cameras that implement an exposure fusion algorithm, will take successive shots of a scene at very short-time intervals, possible mis-registration will be most probably translational. Thus, the chosen MTB method is a very fast and robust approach for correcting mis-registrations in real-time applications.

The next stage of the system was to move the three RGB color channels to another color space, where luminance is separated from chrominance. The YCbCr color system is an invertible color system that maps the RGB channels to a luminance channel Y and two chrominance channels Cb and Cr. The reason behind this action comes from the human visual system. Humans are more sensitive to details/errors that exist in the luminance channel of images and are less sensitive to details in the color channels. The same principle is also used in the JPEG compression scheme, where the chrominance is usually downsampled by a factor of 2, whereas the luminance channel stays intact. That is to say, it is of great importance that the proposed system has great fusion performance in the luminance channel. To satisfy this constraint, we used the Mitianoudis and Stathaki algorithm [4] to fuse the three Y channels from the input images. The Mitianoudis and Stathaki fusion approach is a state-of-the-art algorithm that performs really well at out-of-focus problems. Therefore, it was the optimal choice to fuse the luminance channels of the MEF system. On the other hand, the algorithm’s performance may vary with different intensity-range input images, as in the Cb and Cr channels, due to its dependence on local means choice for the fused image. To offer a more stable solution, the color channels were fused with the Mertens et al. algorithm, which is a more linear blending color solution and offers more valid selection criteria for MEF applications. The next section offers more details in the operation of the two algorithms.

### 2.2. Luminance Channels Fusion

Luminance channels fusion is performed using the Mitianoudis and Stathaki [4] approach. The algorithm is considered a transform-based fusion method. The difference with other transform-based methods is that here the transform, instead of being constant, is learned from data, i.e., similar-content images in our case. Learning is achieved via a statistical adaptive technique, known as ICA. Images are segregated into small local image patches of size N×N. ICA demands several training data (in the order of 10,000 [19]) for a successful transform training. Thus, the algorithm randomly extracts several patches from similar-content training images. For training, these patches do not need to overlap. They just need to be randomly selected from the training images. These N×N patches are then transformed to $N^{2}\times 1$ vectors $x_{w}\left(t\right)$ using lexicographic ordering, i.e., column reordering as a single column. The mean value of each vector is then subtracted. Please note that at this stage, there is no need to store these means, since these patches will solely be used for training.

The basic concept behind this transform learning procedure is to estimate a set of projection (synthesis) bases $b_{i}$ (arranged in matrix $B=[b_{1},b_{2},\cdots ,b_{N}]$) that can yield a sparser representation u(t) of the input image $x_{w}\left(t\right)$.

(1) $$x_{w}\left(t\right)=Bu\left(t\right)$$

(2) $$u\left(t\right)=B^{-1}x_{w}\left(t\right)=Ax_{w}\left(t\right)$$

where $A=B^{-1}$ is the analysis kernel and *t* denotes an increasing patch index. This training procedure is quite computationally demanding, nonetheless, it can be performed only once. The estimated ICA transform is a more general tool that analyze images with similar content to the training images, i.e., it is image-content adaptive but not image-content specific. This implies that it can perform efficiently with a wide variety of images, i.e., it can work with natural images, if it was trained with natural images, which is a great subgroup of images. The learning stages are the following. Principal Component Analysis (PCA) is firstly operated on the selected patches, to perform decorrelation. Additionally, one can also choose the $K<N^{2}$ most important bases using PCA, thus performing dimensionality reduction. Since this step can decrease the system’s quality, by performing compression, it was not selected in our system. The next step is to iterate the ICA update rule in [11] until it converges to a local optimum. In each iteration, the bases are orthogonalized using a Gram-Schmidt orthogonalization. In the case of multimodal inputs, sample patches from all modality inputs are selected to train the ICA bases. ICA has shown to deliver sparse representations of the input data, since it aims at decomposing the observed vectors into statistically independent components, which according to Hyvarinen et al. [20] should follow a highly non-Gaussian statistical profile. For the MEF system, the ICA transform is estimated once and stored for the rest of the analysis.

#### Fusion in the ICA Domain

Once the ICA transform is estimated, it can be applied on image fusion. We extract every possible N×N patch from each input image $x_{k}\left(i,j\right)$. That is to say that we select all the N×N patches of the image with 1-pixel overlap. These are consequently re-arranged to form vectors $x_{k}\left(t\right)$, where *t* is an increasing patch index. We estimate the mean $MN_{k}\left(t\right)$ from these vectors $x_{k}\left(t\right)$, which is then subtracted from the vectors to be normalized to zero mean. The subtracted local means $MN_{k}\left(t\right)$ are stored to be used for the reconstruction of the fused image. The zero-mean vectors $x_{k}\left(t\right)$ are consequently transformed to the ICA-domain representation $u_{k}\left(t\right)$ via Equation (2). Once the vectors are in the ICA domain, optional denoising is also possible here. This is achieved by using a technique called *sparse code shrinkage* on the ICA-domain coefficients [19]. After input denoising, the input vectors in the ICA domain $u_{k}\left(t\right)$ are fused to create the fused image representation $u_{f}\left(t\right)$ in the ICA domain. This fusion process is a method of combining vectors from the different input images to form a single vector. This method is usually called a *fusion rule*.

Once the fused image is created in the ICA domain, the synthesis kernel *B* is used to return the image back to the spatial domain. We also must derive an optimal means $MN_{f}\left(t\right)$ for each patch of the fused image from the local means $MN_{k}\left(t\right)$ of the input images. The gradient rule in [4] is used to estimate an optimal means $MN_{f}\left(t\right)$. When the input images feature similar intensity levels in all images, i.e., an out-of-focus example, the average mean is a very good and simple choice. In these cases, this is usually the result of the gradient rule in [4]. In the case of multimodal inputs, since the inputs have different contrast, the gradient rule of [4] can offer a viable solution. The derived optimal means are then added to the corresponding fused image vectors, which are transformed to image patches. The complete fused image f(i,j) is reconstructed by spatially averaging the image patches $u_{f}\left(t\right)$. The patches are averaged in the same order that were selected during the analysis step.

Many fusion rules have been proposed for ICA-based fusion [11,12]. The fusion by the *absolute maximum* rule selects the greatest in absolute value from the corresponding coefficients in each input vector (“max-abs” rule). This rule seems to detect and transfer all the edge-information from the input images to the fused image. The drawback is that usually constant background areas seem to feature slightly altered intensity. On the other hand, the fusion by *averaging* rule estimates the average of the corresponding input coefficients (“mean” rule). This rule seems to preserve the actual intensity information, especially in background areas. In contrast, the areas of strong edges appear smoother after applying this rule. This is logical since the mean operator acts as a “lowpass” filtering operation in signal processing. Finally, a *Weighted Combination* (WC) rule uses weights $w_{k}\left(t\right)$ to create the fused image using a linear combination of the input ICA representations. These weights emphasize sources with more intense edge activity, in terms of the $L_{1}$ norm. This is equivalent to demand the fused representation to be as sparse as possible, i.e., more well-defined in the ICA bases space. Therefore, we use weight that favor more active patches in terms of the L1-norm, which can be a metric of sparsity. Finally, in [12], Mitianoudis et al. introduced several region-based fusion rules, based on textural information. They used a clustering algorithm to divide the input patches into three categories, according to their content activity: strong edges, texture, and background. A different fusion rule is used for each of three category areas.

In this system, the “max-abs” rule was used, which tends to yield the best results, especially for out-of-focus examples. The Luminance channel is the most important channel in human visual perception in terms of image understanding. According to the information in this channel, humans perceive boundaries and shapes of objects, therefore, it is important that the information in this channel is as sharp as possible. The same strategy is followed in the JPEG standard. Thus, the “max-abs” rule stresses the position of strong edges in the fused image, making it sharper than the original images.

### 2.3. Color Channels Fusion

For the optimal color selection for the fused image, the second fusion algorithm of multiple exposures by Mertens et al. [3] is presented here. This approach fuses the input exposures using simple image quality measurements such as saturation, contrast, and level of good exposure. Thus, the values of quality measures in each pixel of the multiple exposures is estimated. Then, a fusion weight that depends on all three quality measures is estimated for each input image. Finally, a combination of these weights is used to construct a unified set of weights that aims to transfer all useful information from the multiple entry exposures to the fused image. The three quality measures are estimated as follows:

- *Contrast*: a Laplacian edge-detection filter is applied to the grayscale version of each exposure. The grayscale version is estimated by forming a weighted sum of the R,G, and *B* components in the form: 0.2989R+0.5870G+0.1140B. The absolute value of this filter response results in the index *C* that is used to define the image’s contrast. This simple procedure produces large weights on edges and texture, i.e., emphasizing the existence of these image elements.
- *Color saturation*: When a picture gets more exposed, colors tend to lose their sharpness and get more saturated. In this case, the image becomes more vivid, thus saturated colors must be preserved in general. Therefore, we estimate a measurement of color saturation *S*, which is based on the standard deviation of the R, G, and B channels of a neighborhood around each pixel.
- *Well-exposedness*: The intensity channel can highlight whether a pixel is well exposed or not. The main aim of this metric is to retain intensities that are not close to zero (under-exposed intensities) or close to one (over-exposed). Each pixel intensity *x* is weighted by a factor that depends on its distance to 0.5. This is performed by using the Gaussian function curve: $\exp(\left(-{\left(x-0.5\right)}^{2}\right)/\left(2\sigma^{2}\right))$, where σ is usually equal to 0.2. The Gaussian curve is applied independently to both Cb and Cr channels. The two curves are then multiplied to create the well-exposedness measure *E*.

These different metrics *C*, *S*, and *E* are combined into a single weight map for each pixel. This is performed by multiplication to keep the influence of all these factors (similar to a logical and operation). The effect of each metric is controlled via using a power coefficient:

(3) $$W_{ij,k}={\left(C_{ij,k}\right)}^{w_{C}}\times {\left(S_{ij,k}\right)}^{w_{S}}\times {\left(E_{ij,k}\right)}^{w_{E}}$$

where *C*, *S*, and *E* are the contrast, color saturation, and well-exposedness metrics, respectively, and $w_{C},w_{S},w_{E}$ are weighting power factors of the metrics. The indices i,j,k refer to pixel (i,j) of the *k* exposure $x_{k}\left(i,j\right)$. To ensure a sensible and stable result, the estimated weight maps $W_{ij,k}$ are divided by the sum of all weights for the same pixel, i.e.,

(4) $$W_{ij,k}\leftarrow W_{ij,k}/\sum_{k=1}^{K}W_{ij,k}$$

The aim is that all weights for each pixel (i,j) sum up to 1. The *K* input images can be fused via a weighted average for each pixel. The weights for each pixel are given by (3).

(5) $$x_{f}\left(i,j\right)=\sum_{k=1}^{K}W_{ij,k}x_{k}\left(i,j\right)$$

Nonetheless, this simple weighted fusion of the input images may yield undesirable results. In several case, the estimated weights can vary very quickly, which may lead to invalid combinations of the input images that are not useful in terms of exposure fusion and optimal color transfer. To address this problem, one can use the Laplacian pyramid to decompose the input images. The Laplacian pyramid is a multi-scale decomposition that transforms the input image into multiple band-pass filtered versions at different scales of the input. Once, the input images are decomposed using the Laplacian pyramid, the input images at each decomposition level can be separately fused using the weights proposed earlier in this section. Finally, after the pyramid decomposition of the fused image is estimated, the fused image can be reconstructed using the inverse Laplacian pyramid decomposition. Multi-scale fusion is very effective in addressing the aforementioned problem, because fusion is performed at a multi-scale feature level (image edges) instead of raw intensities.

### 2.4. Fused Image Reconstruction and Post-Processing

The two fusion algorithms produce three images: the first represents the fused luminance Y channel while the other two images represent the fused color Cb, Cr channels. The final fused image is reconstructed by transferring these three channels to the RGB system.

In modern HDR applications, one can also encounter a wide variety of post-processing steps that can alter and possibly enhance the color balance of the fused image. These optional steps usually create surrealistic scenes with vivid colors and increased contrast. In the proposed system, we implemented several of these optional post-processing steps. More specifically, we incorporated: (a) edge and detail processing (Highlights and shadows), (b) color enhancement (Auto Color Saturation) and (c) sharpness improvement (Local Contrast Adjustment).

Among several edge and detail processing activities, here we propose edge enhancement and flat area smoothing using *bilateral filtering* [21]. This filtering procedure smoothes images while maintaining their edges with a non-linear combination of close values of the image in contrast to the classical filtering, where the edges appear blurred. This method is non-repetitive, local, and simple. The filter combines gray levels or colors, according to the geometric proximity and their photometric similarity, and prefers nearby values from distant values of both the space and the spectrum. The idea behind the bilateral filtering is to do to the spectrum of an image that traditional filters do in the space. The spatial filtering enforces the proximity attaching to the values of pixels weights that are decreasing with distance. Similarly, a range filter calculates an average of the values with weights decreased by the disparity.

Auto Color Saturation is another additional feature that can be achieved by boosting saturation. There is a patented method that can boost saturation, which is described in [22] in detail. Sometimes, the HDR fused image lack of vivid colors because they do not appear in the input exposures either. We would like to boost the saturation in these cases to produce the desired result. The global increase of the saturation though may lead to forbidden values, so we use a local method to enhance the color of a pixel at time. A determination is made as to whether to reduce or increase the brightness of an image and to what extent to allow the proper amplification of saturation. This is because as the saturation of a color increases, the brightness is automatically decreased. Conversely, the reduction of brightness gives the perception of increased saturation. The brightness adjustment may be done using a lookup table (LUT). Below a lower threshold and above an upper threshold the output pixel intensity is the same with the input one. Between these thresholds, the intensities are adjusted nonlinearly, according to the following equation:

(6) $$I_{out}=I_{in}-\beta \sin\left(\pi I_{in}\right)\;\mathrm{when}I_{L,th}\leq I_{in}\leq I_{H,th}$$

where $I_{in}$, $I_{out}$ are the input and output images respectively, $I_{L,th}$ and $I_{H,th}$ are the lower and higher threshold values and β sets the amount of the adjustment.

To tackle sharpness improvement, a common approach is unsharp masking [23]. Unsharp masking sharpens an image. It cannot create new information but can significantly improve the appearance of details and texture. It uses a blurred version of the original image, which can be obtained with a lowpass/Laplace filter, to locate the edges. This is done via subtraction pixel by pixel. Subsequently, the edge image serves as a mask and the contrast is then increased selectively.

Finally, a series of basic image processing filters can implement the Orton effect. A complete guide on the effect is given in [24]. The effect transforms an image of a real scene in a way that appears more like a painting than a photograph. Thus, it gives a more artistic style to the image. In the original method, two or more photos of the same scene with different properties are required, which are then combined properly resulting an image with pieces of high clarity among blurred colors.

## 3. Experiments

In this section, the performance of the proposed hybrid MEF system is evaluated and compared with other MEF systems.

### 3.1. Methods Compared

The proposed system is compared against five state-of-the-art modern exposure fusion algorithms: (1) Multiple-exposure fusion based on Illumination Estimation (https://sites.google.com/site/vonikakis/software-code) [6], (2) Image fusion via quality metrics (Mertens) [3], (3) Fusion based on bilateral filtering (Raman) [25], (4) Dynamic Photo HDR (https://www.mediachance.com/hdri/), which is a commercial application and (5) Li and Kang method (FMMR) (http://xudongkang.weebly.com) [10]. The proposed system will be referred to as Hybrid HDR, since the fused image is derived by the combination of two different methods. The system’s development and performance evaluation were performed in MATLAB 2015a. The developed system can be downloaded online (http://utopia.duth.gr/nmitiano/download.html) as a MATLAB standalone executable. For the proposed HybridHDR system, 8×8 local patches were used for the ICA fusion framework. No dimensionality reduction and denoising was selected in the ICA framework. Also, no post-processing was added to the fused image, to ensure a fair comparison with the other methods. MATLAB codes for all the compared methods were found online. The source was either the authors’ websites or the MathWorks resource center.

### 3.2. Comparison Metric

The Image Quality Assessment (IQA) model of Ma et al. [26] was used as an evaluation tool of the different algorithms in this work. This metric is based on the principle of the structural similarity approach (known as SSIM [27]) with a novel measure of patch structural consistency. The proposed metric evaluates local structure preservation at fine scales and luminance consistency at coarser scales. This metric gives a maximum score of 1 to the best fusion result, i.e., gives an upper bound to fusion quality in contrast to PSNR type of metrics, which do not have an upper bound.

### 3.3. Dataset and Results

**Figure 2:**
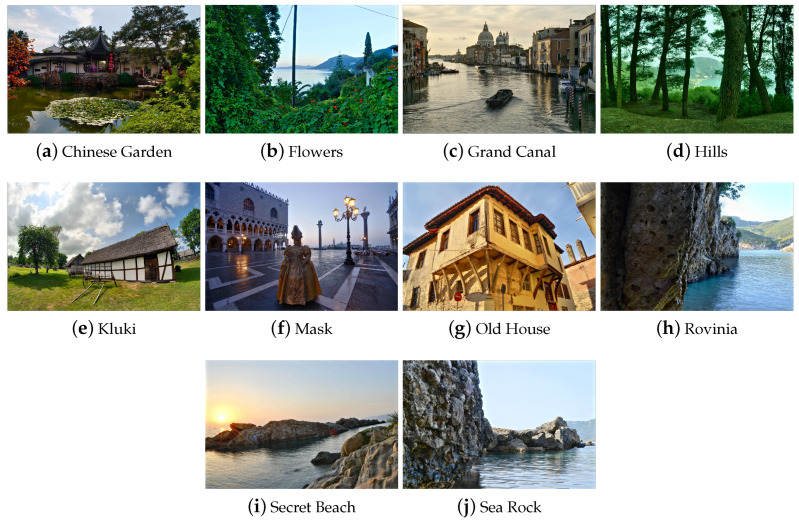
Ten images from various MEF databases and a developed dataset. These ten images were used in our experiments. This figure depicts the results of our method.

For evaluation, 10 sequences of multiple exposures were chosen. These sequences cover diverse visual content, including exterior views, natural landscapes, and buildings. Some of these sequences were selected from several multiple-exposure datasets. To increase experimentation variety, the authors photographed and developed a novel multiple-exposure dataset. The dataset is freely available for download (http://utopia.duth.gr/nmitiano/download.html). The datasets that were used in our experiments are shown in Figure 2. All datasets contain 3 exposures (under-exposure, over-exposure, and normal exposure). Fusion results are evaluated with the Ma et al. [26] metric and are depicted in Table 1.

**Figure 3:**
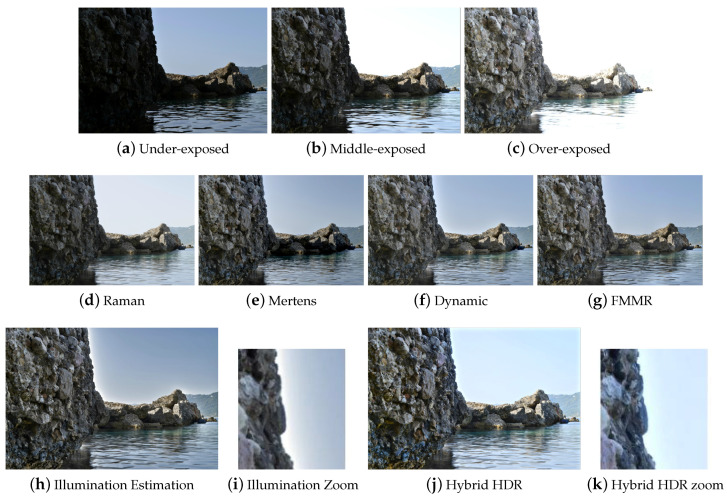
Comparison of the six MEF algorithms using the “SeaRock” images. Halo artifacts remain in the Illumination Estimation method, which are not visible in Hybrid HDR.

**Figure 4:**
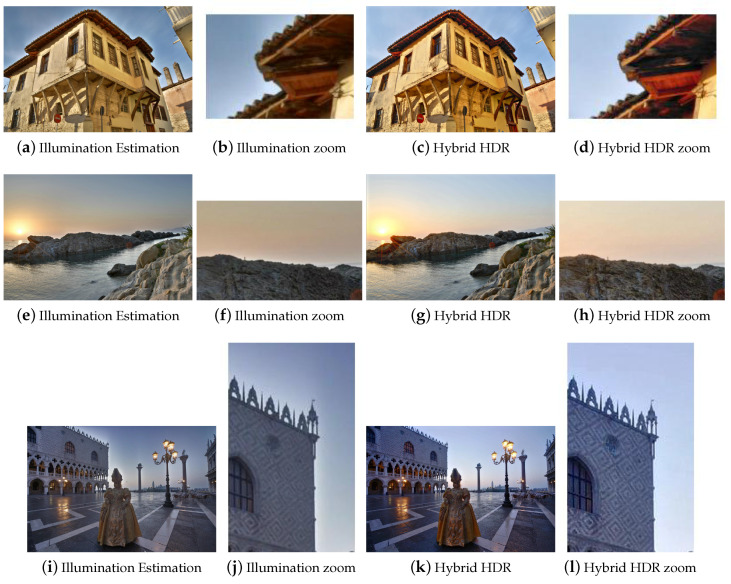
Comparison between the HybridHDR and the Illumination Estimation method for three image sets. More halo artifacts can be spotted in the Illumination Estimation method.

**Figure 5:**
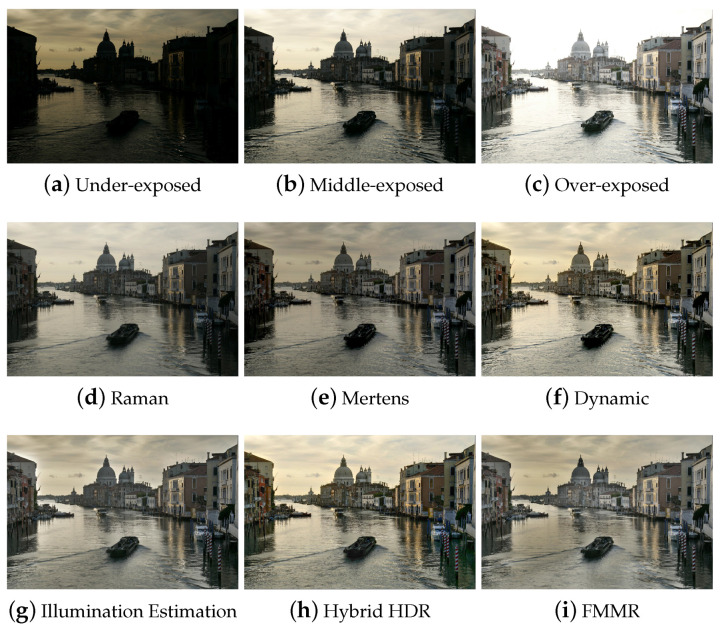
Comparison of the six MEF algorithms using the “Venice” images.

**Figure 6:**
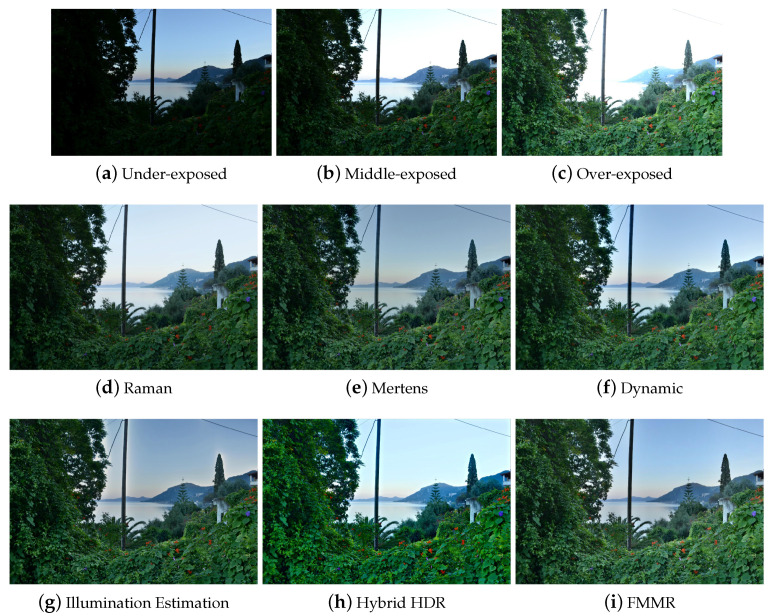
Comparison of the six MEF algorithms using the “Flowers” images.

The results of Table 1 show that the Illumination Estimation method has on average the best score. The proposed Hybrid HDR nearly reaches the best quality. The Dynamic Photo HDR software and Mertens algorithm follow along with the FMMR. The method, which is based on bilateral filtering (Raman) appears to give the worst results. The latter method seems to fail at adequately conveying small details and color information of the input images to the fused image. These conclusions can be verified perceptually by visual inspection of all the results. Some indicative fusion results, depicted in Figure 3, Figure 4, Figure 5 and Figure 6, are adequate to perform subjective evaluation. A promising result is that the proposed HybridHDR performs better than the Mertens method. This implies that using the ICA fusion for the luminance channel complements the traditional Mertens method to produce better MEF results. The FMMR method is a very fast method producing promising results as well.

**Figure 7:**
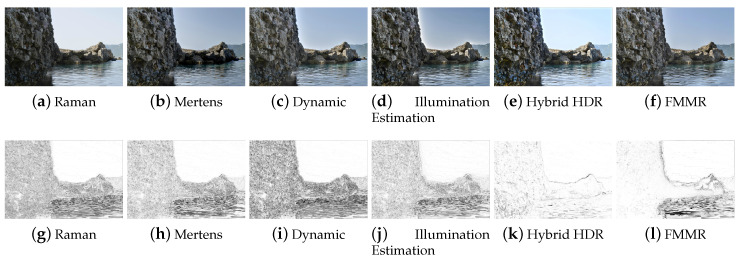
Comparison of the six MEF algorithms in terms of structural similarity. White colors show high structural similarity, whereas dark colors demonstrate low structural similarity.

Figure 7 demonstrate the performance of the six methods in terms of structural consistency of the final image with the three input exposures. Structural consistency maps are estimated using the method proposed in [26]. Lighter areas in the maps indicate areas of high structural consistency of the final image with the three input exposures, while darker areas indicate low structural similarity. The proposed HybridHDR, as well as the FMMR, demonstrate higher structural consistency compared to the other methods. The FMMR seems to be whiter in certain areas but there are strong dark spots, whereas the HybridHDR tends to be whiter, overall.

Finally, we should stress that although the algorithm based on the Illumination Estimation has a better average score than the proposed model (Hybrid HDR), it tends to show artificial objects around the edges (also known as halo artifacts), which results to intense brightness fluctuations (Figure 3, Figure 4, Figure 5 and Figure 6). In these figures, one can easily spot the halo artifacts, especially in the magnified areas. This is because the algorithm does not have a perceptually correct representation of the physical scene at these points creating these artifacts. We reckon that this method shows higher measurements, because the quality assessment metric of Ma et al. does not take account of the brightness components and therefore the effect of the halo artifacts is averaged out as non-important by the metric. In contrast, the proposed Hybrid HDR features the least visible halo artifacts from all produced results, with the most increased contrast. Therefore, we conclude that the proposed method gives, in accordance with the quality metric evaluation of the previous paragraph, better fusion results compared to many state-of-the-art techniques that have been used in our study.

## 4. Conclusions

In this paper, the authors propose a Hybrid multiple-exposure algorithm, by combining a traditional out-of-focus image-fusion algorithm, such as the ICA fusion algorithm, and the Mertens et al. algorithm. The ICA-based image-fusion method is used to fuse luminance input channels, because of its great performance at transferring edges to the fused image. The Mertens et al. is used to fuse color channels Cb and Cr, because it offers more spatially coherent coloring of the fused image. The proposed algorithm outperforms Mertens algorithm in most cases, which is an obvious improvement. The proposed method also scores favorably with the Illumination Estimation method and outperforms the FMMR method.

The authors are looking to expand the proposed method, by ensuring the removal of possible halo artifacts in the fused image. In natural scenes there often appear moving elements, which create halo artifacts, when fused in an HDR image. A possible extension could be to detect such phenomena in the image registration part and inform the image-fusion algorithm about the existence of such areas. The fusion algorithm can select the input pixels from the balanced exposed image only, avoiding creating halo artifacts by fusing all input pixels in the area.

Over the recent years, there has been a lot of research in the field of sparse and overcomplete representations. Thus, an obvious future step will be to replace the complete ICA bases with sparser overcomplete representations learned again from similar-content data. Among the diverse family of sparse learning algorithms [16], there exist the KSVD [15] and the Analysis KSVD [28] as well as the SimCO [29] and Analysis SimCO [30] algorithms that will offer sparser representation compared to the ICA bases and thus sharper fusion results. 

## Figures and Tables

**Table 1:** Objective Quality evaluation for the six multiple-exposure fusion algorithms using the Ma et al. [26] metric.

Method	Illumination Estimation [6]	HybridHDR	DynamicPhoto	Mertens [3]	Raman [25]	FMMR [10]
Flowers	0.9675	0.9720	0.9705	0.9642	0.9064	0.9215
Mask	0.9531	0.9748	0.9777	0.9323	0.9163	0.9335
SeaRock	0.9562	0.9444	0.8822	0.9316	0.8964	0.9133
Paris	0.9643	0.9662	0.9698	0.9522	0.853	0.8935
Sec.Beach	0.9624	0.9381	0.9360	0.9508	0.9270	0.8884
Garden	0.9536	0.9588	0.9724	0.9369	0.9010	0.946
Kluki	0.9605	0.9552	0.9652	0.9601	0.8988	0.9352
Hills	0.9665	0.9671	0.9686	0.9386	0.9166	0.8661
OldHouse	0.9669	0.9770	0.9753	0.9743	0.9591	0.907
SeaCave	0.9706	0.9641	0.9491	0.9336	0.8813	0.9133
Average	0.9621	0.9617	0.9566	0.9474	0.9088	0.9188

## References

[1] Debevec P.E., Malik J. Recovering High Dynamic Range Radiance Maps from Photographs. Proceedings of the ACM SIGGRAPH.

[2] Reinhard E., Stark M., Shirley P., Ferwerda J. Photographic tone reproduction for digital images. Proceedings of the ACM SIGGRAPH.

[3] Mertens T., Kautz J., Reeth F.V. Exposure Fusion. Proceedings of the 15th Pacific Conference on Computer Graphics and Applications.

[4] Mitianoudis N., Stathaki T. (2008). Optimal Contrast Correction for ICA-based Fusion of Multimodal Images. IEEE Sens. J..

[5] Bogoni L., Hansen M. (2001). Pattern-selective color image fusion. Pattern Recognit..

[6] Vonikakis V., Bouzos O., Andreadis I. Multi Exposure Image Fusion Based on Illumination Estimation. Proceedings of the SIPA.

[7] Tico M., Gelfand N., Pulli K. Motion-Blur-Free Exposure Fusion. Proceedings of the 2010 IEEE International Conference on Image Processing.

[8] Jinno T., Okuda M. (2012). Multiple Exposure Fusion for High Dynamic Range Image Acquisition. IEEE Trans. Image Process..

[9] Shen R., Cheng I., Shi J., Basu A. (2011). Generalized Random Walks for Fusion of Multi-Exposure Images. IEEE Trans. Image Process..

[10] Li S., Kang X. (2012). Fast multi-exposure image fusion with median filter and recursive filter. IEEE Trans. Consum. Electron..

[11] Mitianoudis N., Stathaki T. (2007). Pixel-based and Region-based Image Fusion schemes using ICA bases. Inf. Fusion.

[12] Mitianoudis N., Antonopoulos S., Stathaki T. Region-based ICA Image Fusion using Textural Information. Proceedings of the Region-based ICA Image Fusion using Textural Information (DSP2013).

[13] Piella G. (2003). A general framework for multiresolution image fusion: From pixels to regions. Inf. Fusion.

[14] Farid M.S., Mahmood A., Al-Maadeed S.A. (2019). Multi-focus image fusion using Content Adaptive Blurring. Inf. Fusion.

[15] Aharon M., Elad M., Bruckstein A. (2006). K-SVD: An Algorithm for Designing Overcomplete Dictionaries for Sparse Representation. IEEE Trans. Signal Process..

[16] Zhang Q., Liu Y., Blum R.S., Han J., Tao D. (2018). Sparse representation based multi-sensor image fusion for multi-focus and multi-modality images: A review. Inf. Fusion.

[17] Merianos I., Mitianoudis N. A Hybrid Multiple Exposure Image Fusion Approach for HDR Image Synthesis. Proceedings of the 2016 IEEE International Conference on Imaging Systems and Techniques (IST).

[18] Ward G. (2003). Fast, Robust Image Registration for Compositing High Dynamic Range Photographs from Handheld Exposures. J. Graph. Tools.

[19] Hyvärinen A., Hoyer P.O., Oja E., Haykin S., Kosko B. (2001). Image Denoising by Sparse Code Shrinkage. Intelligent Signal Processing.

[20] Hyvärinen A., Karhunen J., Oja E. (2001). Independent Component Analysis.

[21] Tomasi C., Manduchi R. Bilateral filtering for gray and color images. Proceedings of the Sixth International IEEE Conference on Computer Vision.

[22] Sarkar A., Caviedes J., Subedar M. (2013). Joint Enhancement of Lightness, Color and Contrast of Images and Video. U.S. Patent.

[23] Gonzalez R.C., Woods R.E. (2017). Digital Image Processing.

[24] Orton M. (2012). The Orton Effect. http://www.michaelortonphotography.com/ortoneffect.html.

[25] Raman S., Chaudhuri S. Bilateral Filter Based Compositing for Variable Exposure Photography. Proceedings of the Eurographics.

[26] Ma K., Zeng K., Wang Z. (2015). Perceptual Quality Assessment for Multi-Exposure Image Fusion. IEEE Trans. Image Process..

[27] Wang Z., Bovik A., Sheikh H.R., Simoncelli E.P. (2004). Image quality assessment: From error visibility to structural similarity. IEEE Trans. Image Process..

[28] Rubinstein R., Peleg T., Elad M. (2013). Analysis K-SVD: A Dictionary-Learning Algorithm for the Analysis Sparse Model. IEEE Trans. Signal Process..

[29] Dai W., Xu T., Wang W. (2012). Simultaneous Codeword Optimisation (SimCO) for Dictionary Update and Learning. IEEE Trans. Signal Process..

[30] Dong J., Wang W., Dai W., Plumbley M., Han Z. (2016). Analysis SimCO algorithms for sparse analysis model based dictionary learning. IEEE Trans. Signal Process..

